# Differential roles of type I topoisomerases in regulating HPV pathogenesis

**DOI:** 10.1073/pnas.2526296123

**Published:** 2026-01-02

**Authors:** Arushi Vats, Conor W. Templeton, Laimonis Laimins

**Affiliations:** ^a^Department of Microbiology-Immunology, Northwestern University, Chicago, IL 60611

**Keywords:** HPV, topoisomerases, DNA damage, R-loops, life cycle

## Abstract

High-risk human papillomaviruses (HPVs) activate the Ataxia Telangiectasia Mutated and Ataxia Telangiectasia and Rad3-Related pathways by inducing DNA breaks through the action of viral oncoproteins E6 and E7, which target factors such as topoisomerases. Type I topoisomerases cleave and religate a single strand of DNA, and little is known about how they regulate HPV pathogenesis. The levels of type I topoisomerases, TOP1α, TOP3α, and TOP3β, were all elevated in cells maintaining high-risk HPV genomes, as well as in squamous cell carcinomas. Only TOP1α and TOP3β, but not TOP3α, bound to HPV genomes and were critical for regulating viral gene transcription and replication with little effect on cell growth. Furthermore, the knockdown of TOP1α or TOP3β reduced levels of DNA breaks and differentially altered the expression of genes in key pathways. TOP1α knockdown reduced the expression of IL6 and activation of the procytokine signaling pathway. In contrast, TOP3β targeted EGR3, which regulates growth and differentiation. Finally, TOP1α and TOP3β differentially regulate the formation of R-loops, which are critical for viral replication. These findings demonstrate the differential roles of type I topoisomerases in HPV pathogenesis.

## Significance

Our studies show the levels of type I topoisomerases are increased in human papillomaviruses (HPV)-positive cells, leading to enhanced levels of DNA breaks and activation of DNA repair pathways. Furthermore, knockdown of either TOP1α or TOP3β impaired viral replication and transcription. Importantly, these two topoisomerases act by distinct mechanisms to regulate viral functions. Elucidating the mechanisms by which topoisomerases regulate viral gene expression and replication offers a promising avenue for targeted therapeutic intervention.

Human papillomaviruses (HPV) infect epithelial tissues at various body locations, and a subset is responsible for approximately 5% of human cancers (1). Infection by high-risk HPV types is responsible for most cervical and other genital cancers, as well as many oropharyngeal cancers (2, 3). HPVs infect basal keratinocytes that become exposed through microabrasions to establish persistent infections in which viral genomes are maintained as episomes at low copy numbers (4–6). Productive replication or amplification depends upon epithelial differentiation and is restricted to cells in suprabasal layers. Normal cells arrest in G0 as they leave the basal layer for differentiation, while HPV-positive cells remain active in the cell cycle and reenter G2/M phase to allow for production replication, which is referred to as amplification (7–9). The productive life cycle of high-risk HPVs is dependent upon the activation of two DNA damage response pathways: the Ataxia Telangiectasia Mutated (ATM) pathway and the Ataxia Telangiectasia and Rad3-Related (ATR) pathway (10–12). The viral oncogenes E6 and E7 activate these pathways, in large part through the induction of DNA breaks through the increased expression of cellular enzymes such as topoisomerases (13, 14).

Topoisomerases play a critical role in regulating DNA supercoiling, which is essential for various processes, including DNA replication, transcription, and recombination. There are two main classes of topoisomerases: type I topoisomerases include TOP1α, TOP3α, and TOP3β, while type II comprises TOP2α and TOP2β (9, 15). These enzymes alleviate torsional stress by introducing transient breaks in the DNA, allowing the strands to unwind or coil more tightly. Type I topoisomerases (TOP1α and TOP3α/β) cleave a single strand of duplex DNA, while type II topoisomerases (TOP2α and TOP2β) cleave both strands (16, 17). During their catalytic cycle, these enzymes generate covalent DNA–protein intermediates (TOP1cc, TOP2bcc, and TOP3bcc) (18), which are resolved either by the enzymes themselves or through other proteins like TDP1 (tyrosyl-DNA-phosphodiesterase 1) and TDP2 (tyrosyl-DNA-phosphodiesterase 2), working together with DNA repair factors (15, 19). Dysregulation of these processes can lead to DNA damage, genomic instability, and, ultimately, diseases such as cancer. TOP1α can relieve both negative and positive supercoiling stress, while TOP3α and TOP3β can relieve only negative supercoiling (16). Furthermore, TOP3β is the only topoisomerase that can act on both DNA and RNA (20), while TOP3α acts primarily to relieve torsional stress on mitochondrial DNAs (21, 22). TOP1α has been shown to contribute to the regulation of the replication of some viruses, such as SV40, adenovirus, and HSV (23–25), but it remains unknown how other type 1 topoisomerases contribute. Furthermore, knowledge of how topoisomerases contribute to the HPV life cycle is very limited.

Our studies indicate that the cells harboring high-risk HPV episomes or biopsies of high-grade squamous cell carcinomas exhibit substantially elevated levels of type I topoisomerases compared to normal cells. Knockdown of either TOP1α or TOP3β in cells that maintain HPV episomes significantly impairs viral gene transcription and replication, while depletion of TOP3α has no effect. Transcriptome analysis following the knockdown of TOP1α and TOP3β revealed changes in gene expression, particularly the downregulation of the proinflammatory cytokine IL-6 in TOP1α-depleted cells and in TOP3β-depleted cells, the transcription factor EGR3, associated with cell growth and differentiation. In addition, the depletion of TOP1α increased R-loop formation preferentially on viral genomes, while the knockdown of TOP3β enhanced R-loop formation similarly at both viral and cellular regions. These findings identify differential activities of type I topoisomerases that are critical for the regulation of HPV replication and pathogenesis.

## Results

### Levels of Type 1 Topoisomerases Are Increased in HPV-Infected Cells.

**Fig. 1.**
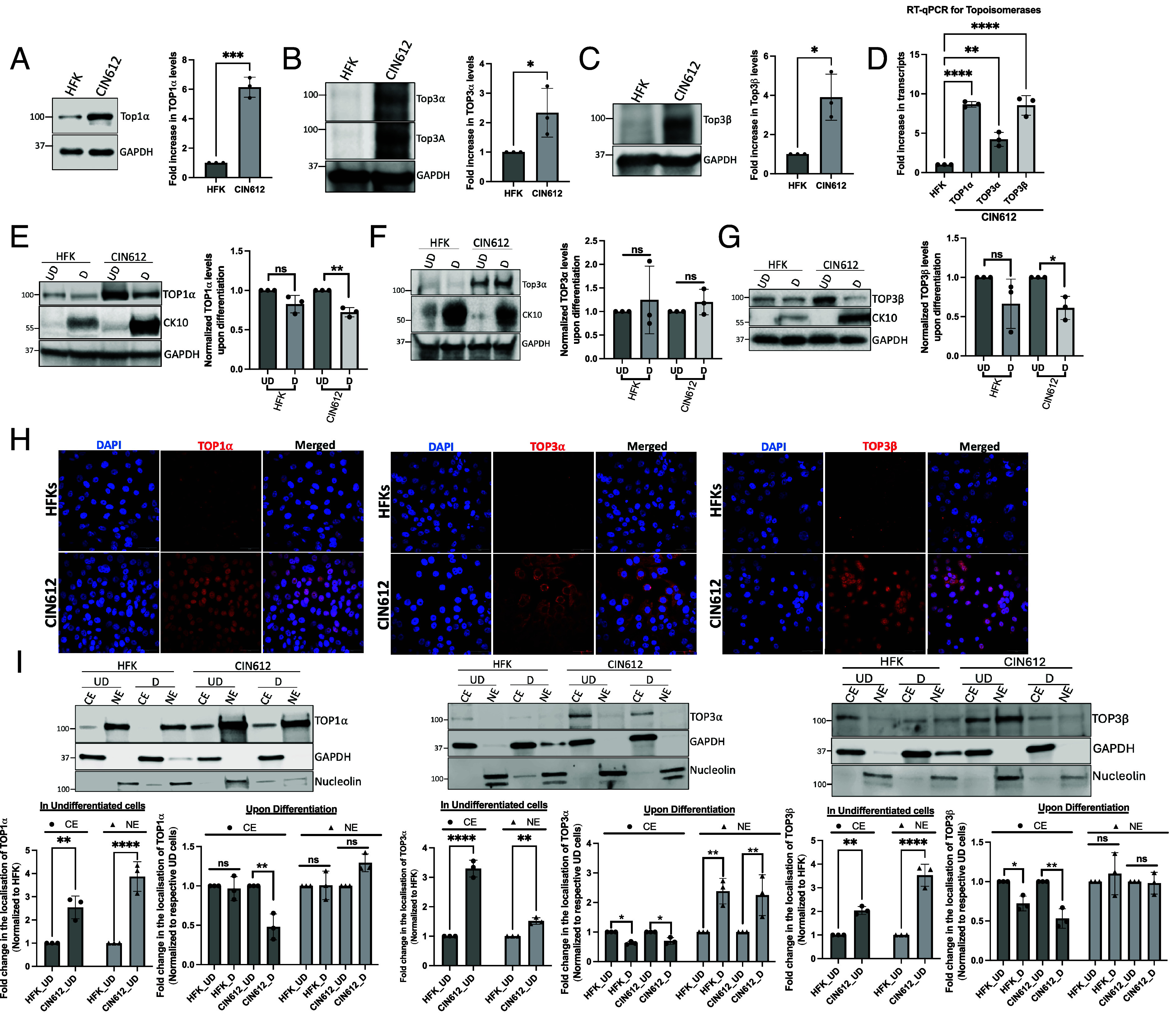
TOP1α, TOP3α, and TOP3β levels are increased in HPV-positive cells. Western blot analysis for TOP1α (*A*), TOP3α (*B*), and TOP3β (*C*) was conducted in HFK and CIN612 cells, with GAPDH used as a loading control (*Left*). The quantification of relative protein expression levels for TOP1α, TOP3α, and TOP3β is normalized to the loading control GAPDH (*Right*). Statistical analysis was performed using a Student’s *t* test in GraphPad Prism; *** indicates *P* < 0.001, and * indicates *P* < 0.05. (*D*) RT-qPCR analysis of TOP1α, TOP3α, and TOP3β transcript levels in CIN612 cells compared to HFK cells, where ** indicates *P* < 0.01 and **** indicates *P* < 0.0001. (*E*–*G*) Western blot analysis for TOP1α (*E*), TOP3α (*F*), and TOP3β (*G*) during differentiation (*D*) is compared to undifferentiated (UD) conditions in HFK and CIN612 cells, with CK10 serving as a differentiation marker. The graphs on the right show quantification normalized to GAPDH, with **P < 0.01 and ns indicating not significant. (*H*) Immunofluorescence analysis shows TOP1α, TOP3α, and TOP3β (red) subcellular localization in HFK and CIN612 cells, with nuclei counterstained by DAPI (blue). Merged images indicate an overlay. (Scale bar, 50 μm.) (*I*) Western blot analysis of cytoplasmic (CE) and nuclear (NE) fractions from UD and differentiated (D) HFK and CIN612 cells probed for TOP1α (*Left*), TOP3α (*Middle*), and TOP3β (*Right*). GAPDH serves as cytoplasmic marker and Nucleolin as nuclear marker. Statistical analysis on the *Left* panels shows subcellular localization of topoisomerases (TOP1α, TOP3α, and TOP3β) in undifferentiated cells. Data show fold change in CE and NE normalized to HFK values for each respective fraction. *Right* panels: Changes in topoisomerase subcellular localization upon differentiation. Data show fold change in differentiated (*D*) samples normalized to respective undifferentiated (UD) controls for each cell line and subcellular fraction. Statistical significance was determined by two-way ANOVA comparing differentiation-induced changes between HFK and CIN612 cell lines within each subcellular fraction. *P < 0.05, **P < 0.01, ***P < 0.001, ****P < 0.0001; ns, not significant. All experiments and statistics are performed from three independent replicates.

To investigate how type I topoisomerases (TOP1α, TOP3α, and TOP3β) contribute to regulating the HPV life cycle, their levels were first examined in HPV-positive cells. For these analyses, the levels of these proteins were examined in CIN612 cells, which are derived from a cervical intraepithelial neoplasia 1 (CIN I) lesion that stably maintains HPV31 episomes and compared to levels present in primary keratinocytes derived from human foreskins (HFK). Western analysis demonstrated a significant increase in the levels of TOP1α, TOP3α, and TOP3β in undifferentiated (UD) HPV-infected CIN612 cells when compared to HFKs (Fig. 1 *A*–*C*). Similarly, RT-qPCR revealed that levels of transcripts were comparably increased (Fig. 1*D*). Given the dependence of the HPV life cycle on epithelial differentiation, the regulation of these topoisomerases was examined following a switch to high-calcium media for 72 h. Switching from low-to-high calcium media induces the differentiation of monolayer cultures of keratinocytes that begins at 24 h and peaks at 72 h, which is also when maximal viral amplification occurs. Western blot analysis of calcium-differentiated CIN 612 cells indicated that levels of TOP1α and TOP3β were present at high levels in comparison to HFKs, though they were moderately decreased from that seen in undifferentiated cells (Fig. 1 *E* and *G*). In contrast, no notable changes were observed in TOP3α levels upon differentiation (Fig. 1*F*). It was next important to investigate whether similar alterations in topoisomerase levels were present in cells with other high-risk HPV types, such as those with HPV 16 and 18 episomes. HFK-16, HFK-18, and HFK-31 cell lines stably maintain viral episomes from high-risk HPVs and were generated through transfection of recircularized cloned viral genomes together with a drug-selectable marker into human foreskin keratinocytes (HFKs), followed by selection and expansion. Elevated expression levels of all three topoisomerases were observed by western analysis of undifferentiated HFK cells harboring episomal HPV-31, -18, and -16 compared to control HFK cells. Upon differentiation, TOP1α and TOP3β protein levels decreased modestly, while TOP3α levels remained unchanged (*SI Appendix*, Fig. S1). Immunofluorescence analysis revealed distinct subcellular localization patterns for each topoisomerase enzyme. TOP1α was predominantly localized to the nucleus, TOP3β showed both nuclear and cytoplasmic distribution, and TOP3α was primarily cytoplasmic in both CIN 612 and HFK cells (Fig. 1*H*). These localization patterns were further validated by cellular fractionation assays (Fig. 1*I*). Notably, fractionation analysis revealed that upon differentiation, TOP1α and TOP3β protein levels decreased specifically in the cytoplasmic fraction with no significant changes in the nuclear fraction, and this pattern was observed in CIN 612 cells but not in HFKs (Fig. 1*I*). In contrast, TOP3α showed a modest decrease in the cytoplasmic fraction, accompanied by a corresponding increase in the nuclear fraction. These findings suggest that the maintenance of high levels of nuclear TOP1α and TOP3β upon differentiation occurs in cells with HPV episomes and stabilized by the action of viral proteins.

### TOP1α and TOP3β, but Not TOP3α, Bind to HPV Genomes and Are Required for Episomal Maintenance.

**Fig. 2.**
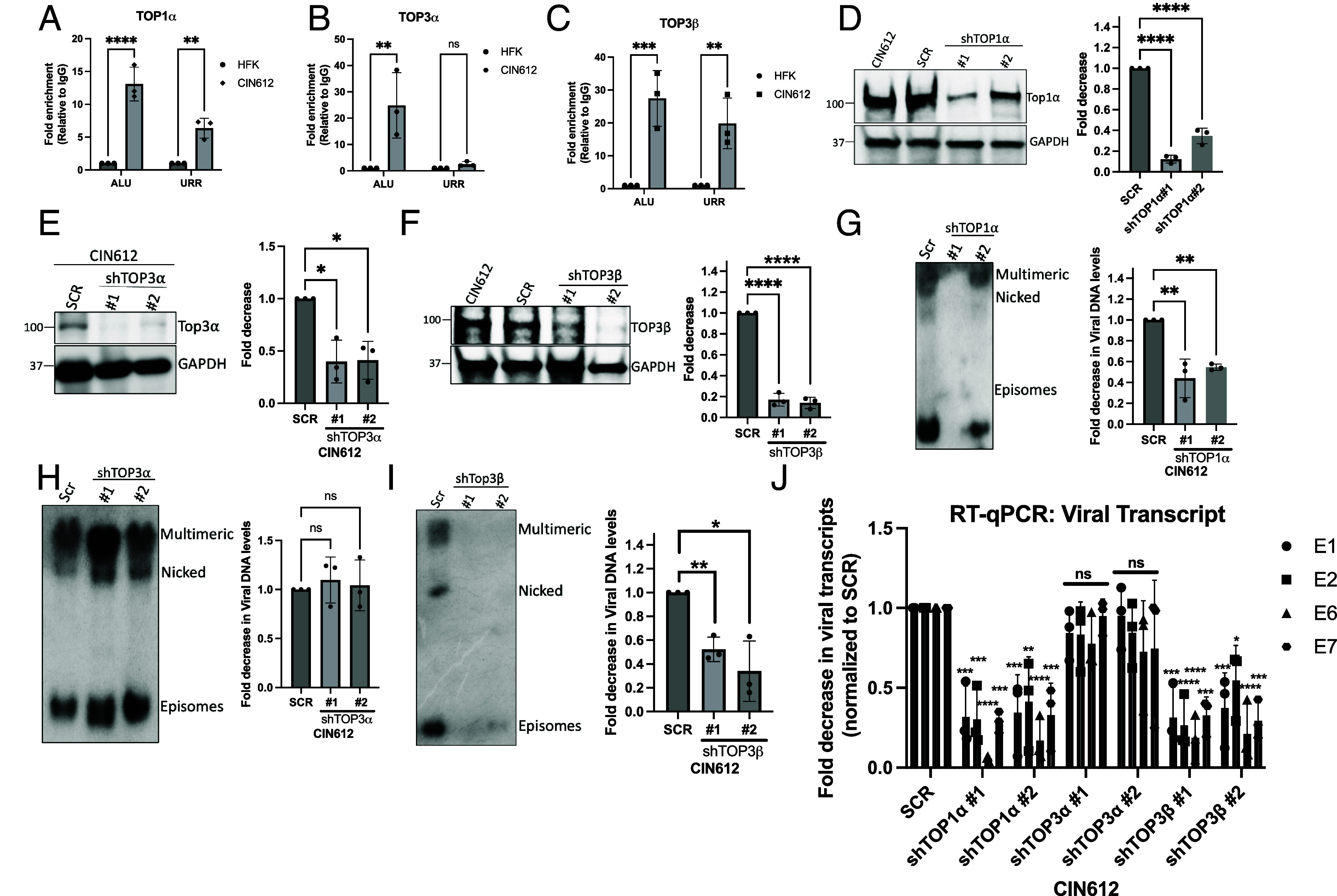
TOP1α and TOP3β bind to the HPV URR region, and their silencing negatively affects the maintenance of the HPV genome. (*A*–*C*) ChIP analysis shows the association of TOP1α (*A*), TOP3α (*B*), and TOP3β (*C*) with the ALU and URR regions in HFK and CIN612 cells in undifferentiated conditions. The data represent fold enrichment relative to the IgG control. ** indicates *P* < 0.01, *** indicates *P* < 0.001, **** indicates *P* < 0.0001, ns indicates not significant. (*D*–*F*) Western blot analysis represents the validation of the knockdown of TOP1α, TOP3α, and TOP3β protein levels from CIN612 cells compared to CIN612, SCR control. GAPDH serves as a loading control. The graph (on the *Right*) depicts the quantification of all the topoisomerases’ knockdown efficiency. ****P < 0.0001, **P < 0.01. (*G* and *H*) Southern blot analysis of HPV DNA forms (multimeric, nicked, and episomal) in TOP1α (*G*), TOP3α (*H*), and TOP3β (*I*) knockdown CIN612 cells compared to SCR control (shown in *Left*). The graph (on the *Right*) shows the quantification of the total viral DNA levels. * Represents *P*-values<0.05. Results are representative of three independent biological replicates. (*J*) RT-qPCR analysis of HPV31 viral transcripts (E1, E2, E6, E7) in CIN612 cells following shRNA-mediated knockdown of TOP1α, TOP3α, or TOP3β. Data shown as fold decrease in viral transcripts normalized to scrambled control (SCR). Error bars represent mean ± SEM. Statistical significance: *P < 0.05, **P < 0.01, ***P < 0.001, ****P < 0.0001; ns, not significant.

Since topoisomerases are known to interact with DNA to regulate supercoiling (16), it was next important to investigate whether these topoisomerases are bound to the viral genome. For this analysis, we performed chromatin immunoprecipitation assays (CHIP) for TOP1α, TOP3α, and TOP3β on HPV 31 upstream regulatory region (URR), which spans the origin of replication, along with ALU repetitive sequences, which were included as a cellular control. Over a 10-fold enrichment of binding of all the topoisomerases (TOP1α, TOP3α, and TOP3β) to ALU sequences in HPV-positive cells was observed in comparison to HFKs. In contrast, only TOP3β and TOP1α (Fig. 2 *A* and *C*), but not TOP3α exhibited binding to the URR (Fig. 2*B*). TOP3α predominantly associates with mitochondrial DNAs (22), and our immunofluorescence indicated it had a largely cytoplasmic localization with only low levels in the nucleus. Interestingly, the binding of TOP3α to ALU repetitive sequences was detected, indicating that some fractions localize to the nucleus. Next, we extended the CHIP analysis using a series of overlapping primers to other regions of the viral genome, including the p97 promoter and the early and late poly-A sites. The p97 promoter exhibited a high association with TOP3β in comparison to TOP1α; however, no significant association was observed with either of the topoisomerases either at early or late poly-A regions (*SI Appendix*, Fig. S2*A*). Collectively, these findings suggest that TOP1α and TOP3β interact prominently at regions of the viral genome crucial for viral replication and the initiation of early transcription.

The elevated levels of topoisomerases found in HPV-infected cells and their association with viral genomes, suggested a potential role in facilitating viral replication and transcription. To examine the effects of topoisomerase depletion on viral genome maintenance and expression, stable knockdown cell lines were generated in CIN612 cells following infection with lentiviruses expressing shRNAs each targeting two distinct regions within TOP1α, TOP3α, and TOP3β. Western blotting and RT-qPCR analyses confirmed a greater than 60% reduction in expression of TOP1α, TOP3α, and TOP3β levels in comparison to scrambled (SCR) shRNA controls for two different shRNA targeting each topoisomerase (Fig. 2 *D*–*F* and *SI Appendix*, Fig. S2*B*).

Southern blot analysis revealed significant decreases in HPV genome levels specifically in TOP1α and TOP3β-depleted cells, while TOP3α knockdown showed no significant effect (Fig. 2 *G*–*I*). Similarly, RT-qPCR analysis demonstrated reduced transcription of early viral genes (E1, E2, E6, and E7) in cells with decreased TOP1α and TOP3β levels, whereas TOP3α silencing had no impact (Fig. 2*J*). These effects were reproducibly observed using two independent shRNA sequences targeting each topoisomerase, confirming the specificity of the observed effects. Given the potential roles of topoisomerases reported in cellular proliferation, we next performed XTT-based cell viability assays to assess the effects of TOP1α, TOP3α, and TOP3β depletion on cell growth. Growth kinetics analysis revealed that all topoisomerase-depleted HPV positive cells exhibited modestly reduced proliferation rates compared to SCR control cells through day 4, after which the growth patterns converged and became comparable to the SCR control (*SI Appendix*, Fig. S2*C*). The convergence of growth rates after day 4 may in part be attributed to cells reaching confluency. In addition, shRNA-mediated knockdown achieved only partial depletion rather than complete elimination of target topoisomerases, as evidenced by detectable residual protein levels in CIN 612 cells following knockdown. This depletion of topoisomerase levels, however, had a pronounced effect on viral replication and transcription without compromising host cell viability. (*SI Appendix*, Fig. S2*C*). These results demonstrate that TOP1α and TOP3β are essential for maintaining HPV genome stability and viral gene expression. Since TOP3α did not associate with viral genomes and knockdown had no effect on viral transcription, our subsequent studies focused on the roles of TOP1α and TOP3β in the HPV life cycle.

**Fig. 3.**
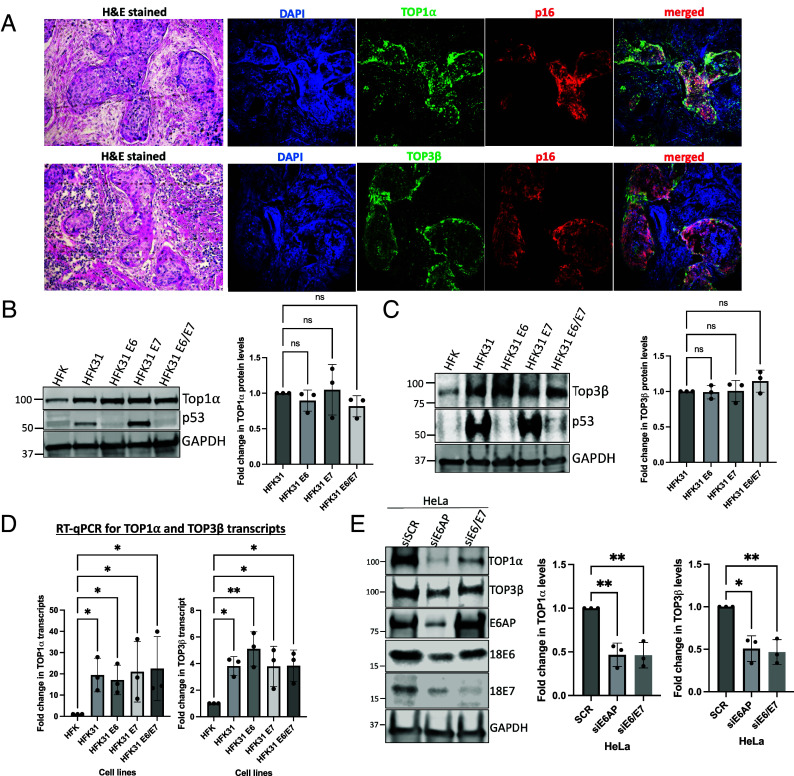
TOP1α and TOP3β levels in cervical cancer tissues and their regulation by HPV oncoproteins. (*A*) Immunofluorescence analysis of TOP1α and TOP3β expression in HPV-positive cervical cancer tissue sections: Representative images showing H&E staining (*Left*) and immunofluorescence analysis of biopsies of cervical cancer tissue stained for TOP1α (*Top*) or TOP3β (*Bottom*) (green), p16INK4a (red), and DAPI (blue nuclei). Merged images demonstrate colocalization patterns in HPV-positive cancer tissue. (*B* and *C*) Western blot analysis of TOP1α and TOP3β in HFK cell lines expressing HPV oncoproteins: Levels of TOP1α (*B*) and TOP3β proteins (*C*) in control HFK cells and HFK cells stably expressing HPV31 E6, E7, or E6/E7 oncoproteins. p53 serves as a control for oncoprotein expression, and GAPDH is included as a loading control. Quantification graphs show fold change normalized to HFK control. (*D*) RT-qPCR analysis of TOP1α and TOP3β transcript levels: mRNA expression levels in HFK cell lines expressing various HPV31 oncoproteins, normalized to HFK control. (*E*) Western blot analysis in HeLa cells following E6AP and E6/E7 knockdown: Levels of TOP1α, TOP3β, E6AP, HPV18 E6, and E7 proteins in SCR control and siRNA-treated HeLa cells (siE6AP and siE6E7). GAPDH serves as loading control. Quantification shows fold change relative to SCR control. Statistical significance: ns (not significant), *P < 0.05, **P < 0.01. Data represent the mean ± SD from three independent experiments.

To investigate whether the increases in TOP1α and TOP3β levels detected in cells that were derived from a precancerous CIN I lesion extended to cervical cancers, immunofluorescence assays were performed on cervical cancer biopsies. Immunofluorescence analysis of tissue sections from HPV-positive squamous cell cervical carcinoma biopsies revealed elevated levels of TOP1α and TOP3β, which correlated with positive p16INK4a immunostaining. p16INK4a serves as a well-established surrogate biomarker for high-risk HPV infection and is overexpressed in the majority of HPV-associated cervical precancers and cancers (26). In contrast to effects in cancerous tissues, both topoisomerases were present at markedly lower levels in adjacent normal cervical tissues, which correspondingly showed minimal or absent p16INK4a expression (Fig. 3*A*; additional replicates are shown in *SI Appendix*, Fig. S3*A*).

To determine whether topoisomerase depletion had any effects on tumor suppressor pathways, we analyzed p53 and pRb protein levels in TOP1α- and TOP3 β-depleted cells. TOP1α knockdown resulted in a greater than twofold increase in p53 protein levels, while TOP3β depletion had no significant effect on p53 expression. Neither topoisomerase knockdown affected pRb protein levels (*SI Appendix*, Fig. S3*B*). This selective p53 upregulation following TOP1α depletion but not TOP3β indicates these enzymes act in different manners to regulate HPV functions.

### Both HPV E6 and E7 Regulate the Increased Levels of TOP1α and TOP3β.

It was next important to identify which viral oncoprotein was responsible for the elevated TOP1α and TOP3β levels, and so western blot and RT-qPCR analyses were performed using HFKs that were stably infected with lentiviruses expressing HPV E6, E7, or both E6/E7 oncoproteins. Expression of E6 alone, E7 alone, or the E6/E7 combination each resulted in significant upregulation of TOP1α and TOP3β at both protein and mRNA levels (Fig. 3 *B*–*D*). These findings were further corroborated in the HPV-18 positive cervical cancer cell line, HeLa, by silencing E6AP ubiquitin ligase using siRNA. E6AP stabilizes E6 and E7, and its depletion significantly reduces levels of these oncoproteins (27–29). Knockdown of either E6AP or direct silencing of E6/E7 both resulted in significant downregulation of TOP1α and TOP3β expression (Fig. 3*E*). Collectively, these results demonstrate that both HPV E6 and E7 oncoproteins contribute to the upregulation of these topoisomerases, suggesting they act coordinately to enhance topoisomerase expression in HPV-positive cells.

### Knockdown of TOP1α and TOP3β Reduces the Formation of DNA Breaks in HPV-Positive Cells.

**Fig. 4.**
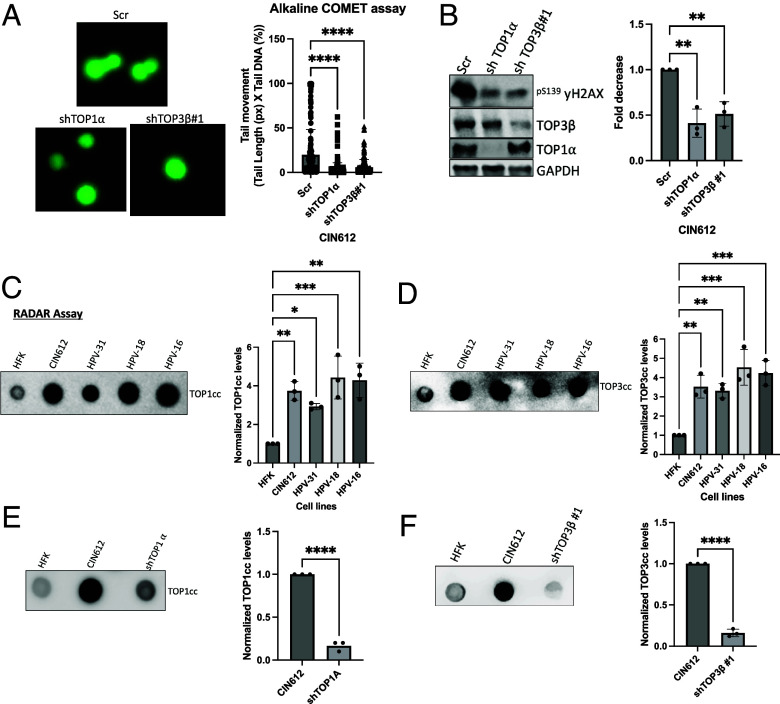
TOP1α and TOP3β induce DNA breaks in HPV- positive cells: (*A*) Representative images of alkaline COMET assay showing DNA damage in CIN612 cells expressing control (Scr), shTOP1α, or shTOP3β constructs (#1) (*Left*). The graph (*Right*) shows the quantification of the tail moment (%). ****P < 0.0001. (*B*) Western blot analysis elucidating the decrease of yH2AX, a DNA damage marker in TOP1α and TOP3β knockdown CIN612 cells compared to SCR control (*Left*) together with the statistical graph showing the quantification of yH2AX protein levels normalized to GAPDH loading control. *** indicates *P* < 0.00. (*C* and *D*) RADAR assay detecting the formation of TOP1α protein-DNA cleaved complexes (Top1cc) (*C*) and TOP3β protein-DNA cleaved complexes (Top3cc) (*D*) in HFK, CIN612, and HFKs stably maintaining episomes for HPV-31, 18, and 16, which decrease in TOP1α (*E*) and TOP3β (*F*) depleted CIN612 cells when compared to SCR control cells. The *Right* panel of the blots represents the statistical significance of TOP1α and TOP3β mediated DNA cleaved complexes in their presence and absence compared to HFKs and SCR control accordingly. All data points represent biological triplicates from independent experiments.

Previous studies have shown that the levels of DNA breaks are twofold to fivefold higher in HPV-positive cells than in normal keratinocytes and that this is responsible for the activation of DNA damage repair (DDR) pathways which is critical for viral replication (30, 31). Topoisomerases can contribute to the induction of DNA breaks through the formation of topoisomerase-DNA cleaved complexes intermediates, which lead to the activation of DDR pathways (32–34). To investigate whether the increase in topoisomerase protein levels results in the increased formation of DNA breaks, alkaline COMET assays were performed to detect both single- and double-stranded DNA damage in TOP1α and TOP3β knockdown cells. In COMET assays, single cells are immobilized in agarose on glass slides, followed by lysis, electrophoresis, and fluorescence imaging. Large intact DNAs remain within the nucleoid body, while fragmented DNAs migrate into the tail region, and the ratio indicates the levels of DNA breaks. Our studies indicate that knockdown of TOP1α and TOP3β in CIN612 cells resulted in an approximately 50% reduction in tail formation compared to control cells (Fig. 4*A*). These results were validated by examining the levels of γH2AX, a marker of DNA damage, by western blot analysis and demonstrated that knockdown of TOP1α and TOP3β leads to a significant reduction in γH2AX levels.

We next investigated whether the increase in topoisomerase protein levels correlated with the increased formation of topoisomerase-DNA cleaved complex intermediates (TOP1cc and TOP3cc) using RADAR assays. A greater than threefold enrichment in the formation of TOP1α and TOP3β mediated cleaved complexes (TOP1cc and TOP3cc) was detected in cells that maintain episomal copies of high-risk HPV types 31, 16, and 18 in comparison to HFKs (Fig. 4 *C* and *D*). The levels of these intermediates decreased significantly upon depletion of TOP1α and TOP3β (Fig. 4 *E* and *F*). In addition to the topoisomerases, tyrosyl-DNA-phosphodiesterase 1, (TDP1 which resolves TOP1cc) and tyrosyl-DNA-phosphodiesterase 2 (TDP2 (which resolves TOP3cc) enzymes can also resolve these DNA–protein complexes (15, 19). To determine whether increased TOP1cc and TOP3cc in HPV-positive cells correlated with changes in TDP1 and TDP2 levels, western blot analysis was performed on HPV-positive cells, and the levels of both enzymes were found to be increased significantly in HPV-positive cell lines relative to HFKs control. Furthermore, knockdown of TOP1α and TOP3β reduced the levels of TDP1 and TDP2, respectively (*SI Appendix*, Fig. S4). These findings indicate that elevated levels of TOP1α and TOP3β contribute to enhanced DNA breaks by increasing the formation of TOP1cc and TOP3cc intermediates, which are subsequently resolved by the cellular enzymes TDP1 and TDP2.

### Depletion of TOP1α and TOP3β Affects the Key Cellular Pathways Involved in HPV Genome Replication.

**Fig. 5.**
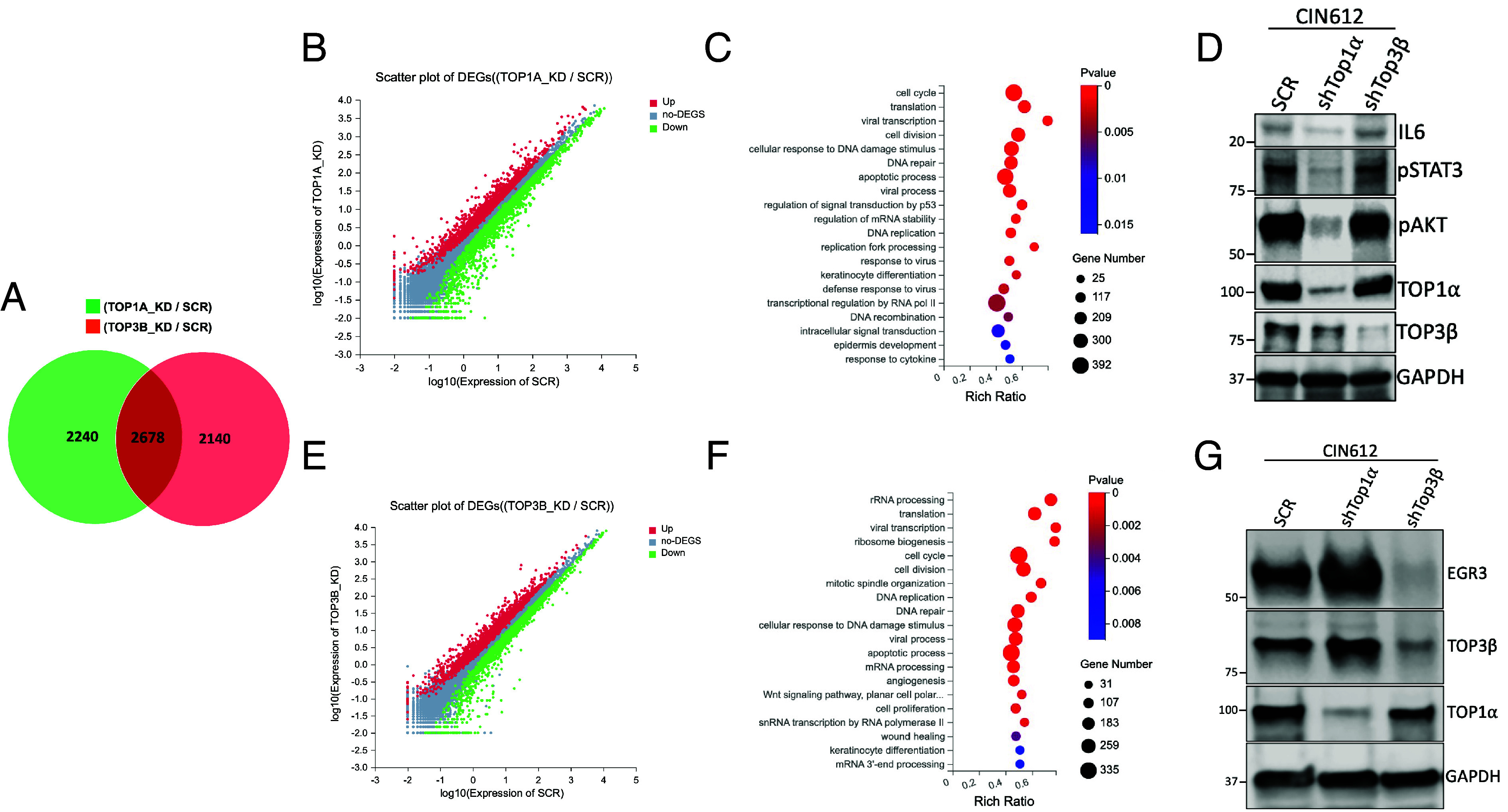
Depletion of TOP1α and TOP3β leads to distinct transcriptional changes. (*A*) A Venn diagram illustrates the overlap of differentially expressed genes between TOP1α and TOP3β knockdown cells compared to the SCR control. (*B*) Scatter plot displays the distribution of differentially expressed genes in TOP1α-depleted cells relative to the SCR control (red: upregulated, green: downregulated, grey: no significant changes). (*C*) Gene ontology analysis reveals enriched biological processes in TOP1α knockdown cells (dot size: gene number, colour gradient: *P*-value significance). (*D*) Western blot analysis shows the levels of IL6, phosphorylated STAT3, phosphorylated AKT, TOP1α, and TOP3β in SCR, shTOP1α, and shTOP3β cells, with GAPDH serving as the loading control. (*E*) Scatter plot displays the distribution of differentially expressed genes in TOP3β-depleted cells relative to the SCR control (red: upregulated, green: downregulated, grey: no significant changes). (*F*) Gene ontology analysis reveals enriched biological processes in TOP3β knockdown cells (dot size: gene number, colour gradient: *P*-value significance). (*G*) Western blot analysis was used to determine the protein levels of EGR3, TOP3β, and TOP1α in SCR, shTOP1α, and shTOP3β cells, with GAPDH serving as the loading control.

Since knockdown of either TOP1α or TOP3β induced a significant reduction in viral transcription, it was important to see whether similar effects on transcription were seen with cellular genes upon depletion of these two topoisomerases. For this analysis, RNA sequencing (RNA-seq) was performed on TOP1α, and TOP3β knockdown CIN 612 cells and compared to HFKs. Our findings identified distinct sets of genes whose expression was substantially altered by the depletion of TOP1α or TOP3β (Fig. 5*A*). Approximately 2,200 genes were uniquely altered in TOP1α or TOP3β knockdown cells, with over 2,600 genes shared between the two. Scatter blot analysis revealed slightly high numbers of genes that were downregulated in TOP1α or TOP3β-depleted cells as they were increased (Fig. 5 *B* and *E*) (Datasets S1 and S2). Importantly, the pathways targeted by the knockdown of these topoisomerases were different. In TOP1α-depleted cells, alterations in cellular pathways essential for the HPV life cycle, such as cell cycle control, DDR, DNA recombination, and innate immune response, were found to be targeted (Fig. 5*C*). Furthermore, a significant reduction in IL-6 expression, a proinflammatory cytokine, was observed upon TOP1α knockdown, which was not seen in TOP3β knockdown cells. IL-6 is known to activate STAT3 and AKT signaling pathways through their phosphorylation (35–37). Consistent with the decreased IL-6 levels, a reduced level of phosphorylation of both STAT3 (pSTAT3) and AKT (pAKT) was detected by western blot analysis in TOP1α knockdown cells but not upon TOP3β knockdown, which retained normal phosphorylation levels of these proteins (Fig. 5*D*). Phosphorylated STAT3 and AKT are known to enhance IL-6 expression in cells (38), suggesting a mechanistic link with TOP1α depletion. In contrast, TOP3β-depleted cells exhibited significant changes in the expression of genes in pathways crucial for RNA processing, DNA repair, and recombination (Fig. 5*F*). In particular, a significant reduction (over fivefold) in the expression of the early growth response 3 gene (EGR3), a transcription factor that plays a critical role in cell growth and differentiation (39), was detected only in TOP3β knockdown cells (Fig. 5*G*). These studies indicate that TOP1α and TOP3β act to regulate different sets of genes that are each critical for HPV pathogenesis.

**Fig. 6.**
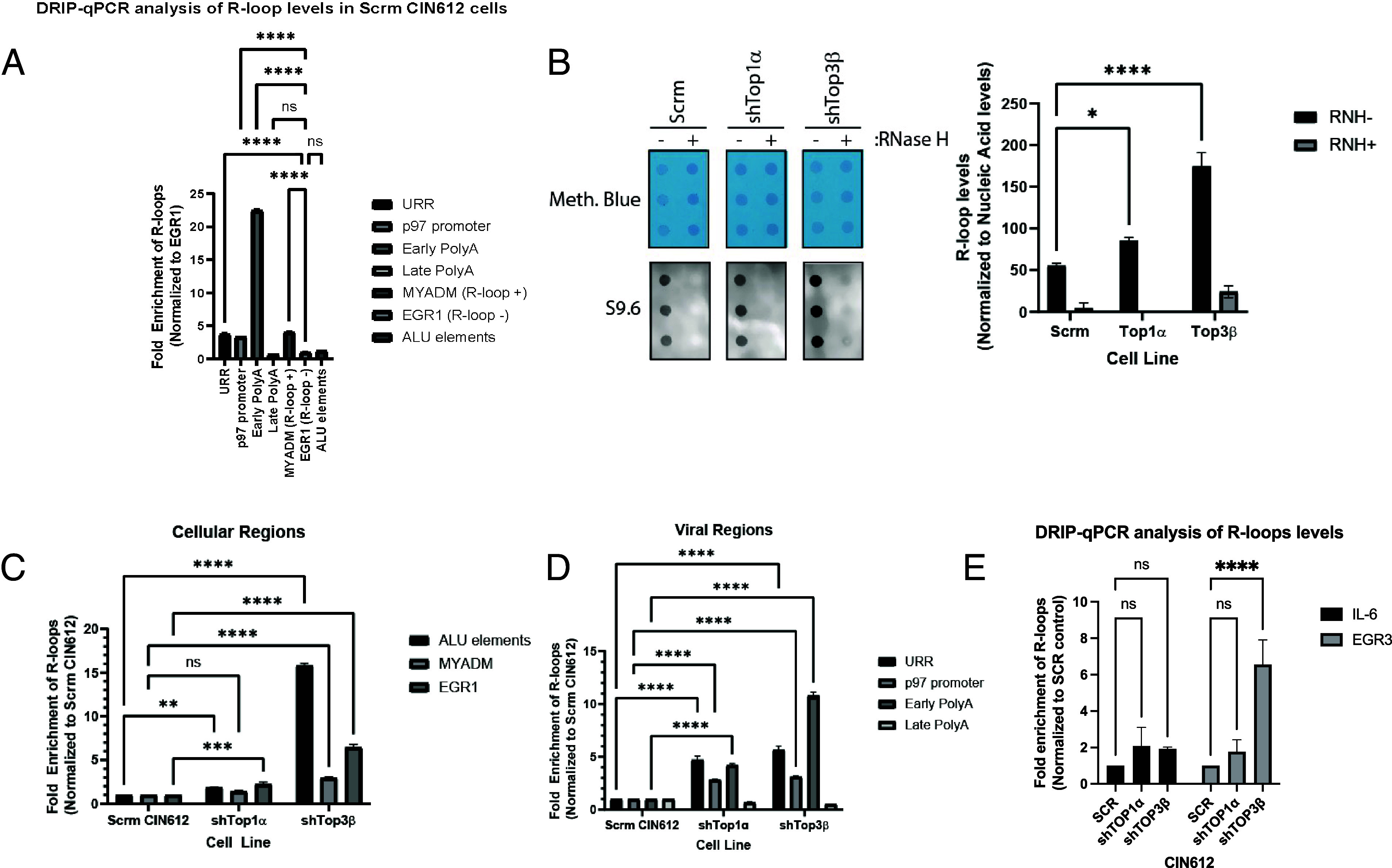
TOP1α and TOP3β regulate R-loop formation at both viral and cellular regions: (*A*) DRIP-qPCR analysis demonstrates baseline R-loop levels across various genomic regions, including viral (URR, p97 promoter, Early PolyA, Late PolyA) and cellular regions (MYADM, EGR1, ALU elements) in CIN612 cells, normalized to EGR1. (*B*) S9.6 dot blot analysis of R-loops in SCR, shTOP1α, and shTOP3β cells with and without RNase H treatment. The *Upper* panel displays methylene blue staining as a loading control, while the *Lower* panel presents the S9.6 antibody signal. R-loop levels normalized to nuclear acid levels are quantified on the *Right*. (*C*) DRIP-qPCR analysis of R-loop formation at viral regions (URR, p97 promoter, early PolyA, and late PolyA) in SCR, shTOP1α, and shTOP3β cells. (*D*) DRIP-qPCR analysis of R-loop formation at cellular regions (ALU elements, MYADM, and EGR1) in SCR, shTOP1α, and shTOP3β cells. (*E*) Gene-specific DRIP-qPCR analysis indicates R-loop formation at IL-6 and EGR3 loci in SCR, shTOP1α, and shTOP3β cells. Data are presented as fold enrichment normalized to SCR control. Statistical significance is noted as ns (not significant), *P < 0.05, **P < 0.01, ***P < 0.001, ****P < 0.0001. Results are representative of three independent biological replicates.

TOP1α and TOP3β have also been implicated in resolving R-loop structures (40, 41). R-loops are trimeric nucleic acid structures consisting of a hybrid between RNA and its complementary DNA strand along with the displaced single-strand DNA (42, 43). R-loop levels have been shown to be elevated significantly in HPV-positive cells and form on HPV episomes at the URR and early polyA (Fig. 6*A*). Previous studies further demonstrated that R-loop formation is critical for viral transcription and replication (44). To assess the impact of TOP1α and TOP3β knockdown on R-loop formation in HPV-positive cells, dot blot assays (DRIP assays) were conducted using the S9.6 antibody, which specifically detects R-loops. DRIP assays showed that TOP1α depletion increased R-loop formation by approximately 50%, while TOP3β knockdown resulted in a more substantial increase of over threefold. Both of these increases were reduced with RNase H treatment, demonstrating the effects were specific for R-loops (Fig. 6*B*). Examination of viral regions at which R-loops form demonstrated that depletion of TOP1α and TOP3β significantly enhanced R-loop formation at the URR and p97 promoter sites, with TOP3β knockdown showing particularly strong effects at the early polyA site (Fig. 6*C*). With respect to cellular genes, TOP3β depletion led to widespread increases in R-loop formation across ALU elements, MYADM, and EGR1, while TOP1α depletion had more modest effects (Fig. 6*D*). Knockdown of either TOP1α or TOP3β exhibited nonsignificant R-loop levels at the IL-6 locus, compared to SCR control. In contrast, a significant increase of over sixfold in R-loop levels was detected at the EGR3 gene that was specific for TOP3β-depleted cells (Fig. 6*E*).

RNA-seq analysis also revealed a slight reduction (0.5 fold) in the expression of enzymes involved in regulating RNA biogenesis, including DHX9, an R-loop resolving enzyme, and POLR3H, a subunit of the POL3 complex necessary for regulating R-loop formation (45–47), following TOP3β knockdown. Despite modest reductions at the mRNA level, we observed no significant changes in protein levels in either TOP1α or TOP3β knockdown cells (*SI Appendix*, Fig. S5*A*). However, coimmunoprecipitation pulldown assays interestingly showed the interaction of POLR3H specific to TOP3β, while DHX9 interacts with both TOP1α and TOP3β, though more significantly in TOP3β-depleted cells (*SI Appendix*, Fig. S5*B*). R-loops specific pulldown revealed a decreased association of these enzymes with R-loops in the absence of TOP3β, suggesting that TOP3β may play a critical role in facilitating the recruitment of both DHX9 and POLR3H to R-loop structures (*SI Appendix*, Fig. S5*C*). This finding indicates that while TOP3β does not affect the overall expression of these R-loop regulatory enzymes, it substantially influences their functional interaction with R-loops, potentially explaining the dramatic increase in R-loop accumulation observed following TOP3β depletion. Overall, these findings suggest that while both TOP1α and TOP3β regulate R-loop formation, they operate through distinct mechanisms: TOP3β functions more broadly across viral and cellular regions, potentially through the recruitment of R-loop resolving enzymes, while TOP1α’s effects are preferentially concentrated in viral regions.

## Discussion

Topoisomerases provide critical functions in resolving torsional stress in DNAs by inducing DNA breaks and are critical for regulating several cellular processes (15). Our study offers insights into the distinct roles of type 1 topoisomerases in regulating the HPV life cycle through distinct molecular pathways. The levels of all three types I topoisomerases (TOP1α, TOP3α, TOP3β) are increased by up to sixfold in HPV-positive cells over those in normal cells. These increases are not the result of replicating viral episomes or other viral proteins, as similar induction is seen by expression of the viral oncoproteins E6 and E7 alone. Western blot and RT-qPCR analyses demonstrated that expression of E6 alone, E7 alone, or both resulted in significant upregulation of TOP1α and TOP3β at protein and mRNA levels, while silencing E6AP or E6/E7 in HeLa cells reduced their expression. Furthermore, these increases are also seen in biopsies of squamous cell carcinomas that can contain integrated copies of HPV genomes, where immunofluorescence analysis revealed elevated TOP1α and TOP3β levels that correlated with p16INK4a staining, compared to adjacent normal cervical tissues.

Our studies demonstrate that only two type I topoisomerases, TOP1α and TOP3β, act to regulate HPV pathogenesis, and they function in distinct ways. Both TOP1α and TOP3β were found to stably associate with HPV genomes at the URR, which contains the origin of replication (Ori) and overlaps the promoter for the early viral gene expression. In contrast, while TOP3α was found to bind to ALU sequences, it did not show any association with the URR or any other region of the viral episomes. This differential binding with the viral episomes correlates with the contributions of these enzymes to the regulation of viral functions. Knockdown of TOP1α and TOP3β resulted in significant impairment in the replication of viral episomes as well as reduced viral gene expression, while depletion of TOP3α had no effect on these functions. Although these topoisomerases play critical roles in cellular processes, their knockdown had minimal effects on the growth pattern of HPV-infected cells, contrasting with reports of TOP1α effects in other cell types (48). Growth kinetics analysis revealed that topoisomerase-depleted cells exhibited modestly reduced proliferation rates compared to control cells through day 4, after which growth patterns converged and became comparable to controls. Furthermore, immunofluorescence revealed that both TOP1α and TOP3β are localized primarily within the nucleus of the cells, while TOP3α is present mostly in the cytoplasm. Interestingly, while there was minimal localization of TOP3α to the nucleus as depicted by immunofluorescence, a small fraction must be present as it was found to associate with ALU1 repetitive elements, though it had no effect on viral pathogenesis. Interestingly, fractionation analysis showed that upon differentiation, TOP1α and TOP3β levels decreased in the cytoplasmic fraction while nuclear levels remained stable in HPV-positive cells, further underscoring their importance in HPV genome replication (22).

The enhanced levels of TOP1α and TOP3β resulted in increased formation of DNA breaks, which leads to the activation of DNA repair pathways (33, 34, 49). HPVs require activation of the ATM and ATR pathways for viral replication and transcription (6, 50) and may contribute to TOP1α and TOP3β effects on viral replication. Both DNA break formation, as determined by COMET assays, and an increase in the damage marker yH2AX levels were detected following the knockdown of either topoisomerase. Topoisomerases generate DNA breaks by forming topoisomerase-mediated DNA-cleaved complexes, which are repaired by the enzymes TDP1 and TDP2 (18). If these breaks are unresolved, they can be detrimental to cells (18). RADAR analysis elucidated a significant increase in TOP1α and TOP3β mediated DNA cleaved complexes (TOP1cc and TOP3cc) in high-risk HPV-positive cells. These increases in repair enzymes TDP1 and TDP2 protein levels are consistent with a high degree of break formation in HPV-positive cells.

Our studies examined the expression of type I topoisomerases in a cell line derived from a CIN I or low-grade squamous intraepithelial lesion (LSIL) and found similar changes to those in cervical cancer biopsies. Future studies examining expression in a range of biopsies, ranging from LSIL to cancer, can provide additional important insights. The most significant differences in the action of TOP1α and TOP3β in HPV-positive cells were in the spectrum of genes whose expression was altered by knockdown. The silencing of TOP1α affects the expression of genes regulating DNA damage and innate immune responses with a significant effect on the IL-6 signaling cascade, which correlated with decreased activation of STAT3 and AKT by phosphorylation. The activation of STAT3 and AKT pathways also regulates IL-6 expression, and the decreased pAKT or pSTAT3 levels observed in TOP1α-depleted cells may also contribute to the downregulation of IL-6 (35, 38). The reduction in IL-6 expression following TOP1α depletion is particularly significant given the established role of HPV E6 and E7 oncoproteins in modulating STAT signaling pathways. E6 and E7 are known to interfere with interferon and cytokine responses, and the TOP1α-mediated regulation of IL-6 may represent an additional mechanism by which HPV modulates the inflammatory microenvironment necessary for viral persistence. In contrast, TOP3β knockdown had no effect on IL6 but rather altered expression of genes involved in RNA processing, DNA repair, and cell growth pathways with significant effects on EGR3 expression, which may be linked to R-loop regulation. The downregulation of the transcription factor EGR3 following TOP3β depletion provides insight into the growth effects we observe. EGR3 regulates cell cycle progression and differentiation pathways. As mentioned, TOP3β knockdown is not complete, and the residual levels of EGR3 appear sufficient for growth but may have effects on viral replication as well as viral and cellular gene expression. The sixfold increase in R-loops at the EGR3 promoter following TOP3β knockdown suggests a potential direct mechanistic link. Similarly, TOP1α knockdown resulted in selective p53 upregulation but not with TOP3β knockdown, indicating these enzymes regulate HPV functions through different mechanisms. Both TOP1α and TOP3β have been shown to regulate R-loop formation; however, they appear to target different sets of R-loops. Topoisomerases negatively regulate R-loop formation by resolving supercoiling ahead of RNA polymerase II and their depletion leads to persistent torsional stress, causing R-loop accumulation across the genome, even in areas lacking R-loop resolving factors or in inactive chromatin (51, 52). Importantly, topoisomerase activity itself induces transient DNA strand breaks (53), which may likely explain the significant increase in DNA damage seen in HPV-infected cells in their presence, independent of R-loop-related genomic instability. TOP1α, however, appears to act preferentially at R-loops on HPV genomes, particularly those at the early polyA, while TOP3β acts equally at both viral and cellular sites. Furthermore, TOP3β depletion induces a sixfold increase in R-loops at the EGR3 promoter locus, which is not seen with TOP1α knockdown. TOP3β helps in the recruitment of R-loop resolving enzymes DHX9 and RNA processing enzyme POLR3H to R-loops, and our studies indicate its absence impairs their association with these factors, contributing to R-loop accumulation following TOP3β depletion.

Our studies also indicate that TOP1α and TOP3β do not form stable complexes at all R-loops but only subsets, while at others, they only associate transiently or indirectly. HPV has two major regions at which R-loops form: the URR/early promoter and the early poly A /termination region (44). While both topoisomerases form stable complexes at the URR regions, this is not seen at the other despite the fact knockdown of TOP3β results in increased levels of R-loops at this site. This could be due to indirect effects or transient association. TOP1α has been reported to bind to the viral E2 protein, facilitating its binding to viral genomes, which may explain its stable association at the URR (54, 55).

**Fig. 7.**
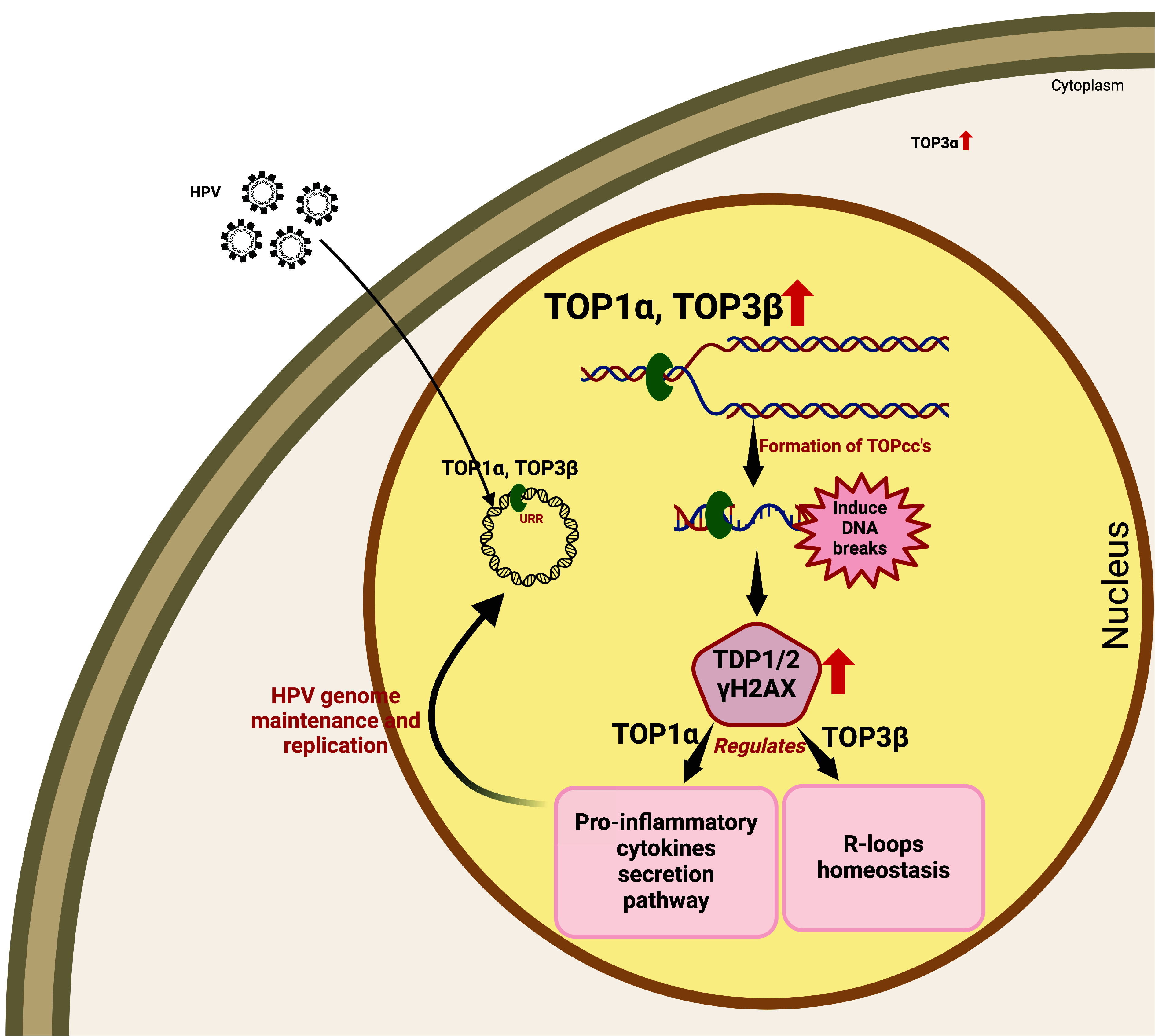
Graphical representation depicting differential roles of TOP1α and TOP3β in regulating the HPV life cycle.

Previous studies examined the effects of type II topoisomerase TOP2β on viral replication and found that it also plays a critical role. TOP2β is part of the CTCF/SMC1 complexes, which form to resolve the tension generated by DNA loop formation (56). Our previous studies, along with others, showed that these factors were enhanced in HPV-positive cells, resulting in DNA looping that regulates viral gene expression (49). TOP2β acts through various pathways to regulate viral functions (57). While TOP1α has been recognized as crucial for replication in several viruses, including HSV, SV40, adenovirus, and HIV (23, 24, 58, 59), its role in the HPV life cycle remained unexplored, and the functions of TOP3α and TOP3β in viral regulation had not been investigated. Our research reveals that both TOP1α and TOP3β, but not TOP3α, are essential for HPV replication and gene expression. This study provides evidence of TOP3β’s involvement in viral regulation and demonstrates that these enzymes operate through different molecular mechanisms to support viral persistence (shown in Fig. 7). These insights enhance our understanding of virus–host interactions and may inform future therapeutic strategies for HPV-associated malignancies.

## Materials and Methods

### Cell Culture.

Primary HFKs were isolated from deidentified neonatal foreskin specimens obtained from the Skin Disease and Research Core facility at Northwestern University. The isolation procedure involved mincing the foreskin tissue into fragments, followed by enzymatic digestion overnight at 4 °C in a Dispase solution. The epidermis was then separated and mechanically dissociated using forceps in a trypsin solution to create a single-cell suspension. Cells from CIN612, which maintain episomal HPV31 genomes, were derived from a deidentified cervical intraepithelial neoplasia biopsy. Both HFKs and CIN612 cells were maintained in E-media as described previously (60, 61), with Mitomycin C-treated NIH-3T3-J2 fibroblasts (J2s) acting as feeder cells at 37 °C in a humidified incubator containing 5% CO_2_. HEK293T and HeLa cells were cultured in DMEM supplemented with 10% FBS and 1% Penicillin-Streptomycin. Stable HFK cell lines harboring HPV31 episomes and those expressing the viral oncoproteins E6 and E7 were generated as previously outlined (62).

To create topoisomerase-knockdown cell lines, CIN612 cells were transduced with lentiviral vectors containing specific shRNAs that target TOP1α, TOP3α, or TOP3β. The transduced cells were subsequently selected using puromycin at a concentration of 4 μg/mL for 3 wk and maintained in E-media with mitomycin C-treated J2 fibroblast feeders.

### Antibodies and Plasmids.

The primary antibodies used included anti-rabbit TOP1α (Bethyl Laboratories # 50-156-2967), anti-rabbit Top3α (Thermo Fisher # 14525-1-AP), anti-mouse Top3β (Sigma Aldrich # WH0008940M1), anti-mouse GAPDH (Santa Cruz # sc-47724), anti-rabbit IL-6 (Cell Signaling Technology, #12912), anti-mouse CXCL10 (Thermo Fisher # MA1-80897), anti-rabbit EGR3 (Cell Signaling Technology, #2559), anti-rabbit yH2AX (S139) (Cell Signaling Technology, #9718), anti-rabbit TDP1 (Abcam, # ab227144), anti-rabbit TDP2 (Bethyl Laboratories, # 50-156-3345), anti-mouse CK10 (Santa Cruz, # sc-52318), anti-mouse p53 (Santa Cruz, # sc-126), anti-mouse E6AP (BD Biosciences, # 611416), anti-mouse 18E6 (santa cruz, # sc-365089), anti-mouse 18E7 (santa cruz, # sc-365035), anti-mouse pRb (Thermo Fisher, # BDB554136), anti-mouse p16 INK4A (BC42) (Cell Signaling Technology, #68410) anti-rabbit pSTAT3 (Abcam, # ab32143), anti-rabbit pAKT (Cell Signaling Technology, #4075), anti-rabbit POLR3H (Thermo Fisher, # 16423-1-AP), anti-rabbit DHX9 (Thermo Fisher, # 17721-1-AP), and anti-mouse S9.6 (Sigma Aldrich, #MABE1095). The secondary antibodies used were anti-mouse IgG, HRP-linked Antibody (Cell Signaling Technology, # 7076S), and anti-rabbit IgG, HRP-linked Antibody (Cell Signaling Technology, #7074S). Predesign shRNA plasmids for the knockdown of each topoisomerase were ordered from Sigma Aldrich. Sequences are provided below:

TOP3β: #1 CCCTGAGAACTTTGACCTGAA (Sigma Aldrich, TRCN00000049313);

#2 CCCTGTGCATATCAACAACAT (Sigma Aldrich, TRCN00000049314)

TOP3α: #1 GCCAGAATGTTACCATGGTAA (Sigma Aldrich, #TRCN00000049295);

#2 GCTTCTCGAAAGTTGAGAATA (Sigma Aldrich, #TRCN00000049297)

TOP1α: #1 TGAAGGGCGAGTGAATCTAAG (Sigma Aldrich, #TRCN0000003987);

#2 GCTTCTCTAGTCCACCACAAA (Sigma Aldrich, # TRCN0000003991)

### Generation of Topoisomerase Knockdown Stable CIN612 Cell Lines.

Lentiviral vectors containing short hairpin RNA (shRNA) sequences targeting TOP1α, TOP3α, and TOP3β were produced using the HEK293T packaging cell line. HEK293T cells were cotransfected with the respective shRNA-containing transfer plasmids, packaging plasmids (psVSVG), and envelope plasmids (pMD2.G) using Lipofectamine 2000 (followed the manufacturer’s protocol). Following transfection, cells were incubated at 37 °C in a humidified atmosphere containing 5% CO_2_. Viral supernatants were harvested at 48 h posttransfection, filtered through 0.45 μm pore-size filters to remove cellular debris, and used immediately for transducing CIN612 cells. For transduction, CIN612 cells were seeded at approximately 30 to 40% confluence in 6-well plates and cultured in E-media supplemented. Cells were transduced with the respective lentiviral supernatants in the presence of 8 μg/mL polybrene to enhance transduction efficiency. Following a 48-h incubation period to allow for viral integration and initial protein expression, transduced cells were subjected to antibiotic selection using puromycin at a concentration of 4 μg/mL. Selection was maintained for 2 to 3 wk with regular media changes every 2 to 3 d to eliminate nontransduced cells and establish stable knockdown populations. The efficiency of topoisomerase knockdown in the selected cell populations was validated using both western blot analysis and RT-qPCR.

### Calcium-Induced Differentiation.

On day 1, cells were seeded at a density of 1.5 × 10^^6^ cells in a 6-well plate and 5 × 10^^6^ cells in p10-cm dishes containing M154 media supplemented with 0.07 mM CaCl_2_ and human keratinocyte growth serum (HKGS) (LifeTech, #S0015). On day 2, the media was changed to M154 with 0.03 mM CaCl2 and HKGS, followed by incubation for another day. On day 3, the media was replaced with M154 containing 1.5 mM CaCl_2_ without HKGS, and the cells were incubated at 37 °C for 72 h before being harvested for further analysis.

### Nuclear and Cytoplasmic Cellular Fractionation.

For nuclear and cytoplasmic extraction (CE), 5 × 10^^6^ undifferentiated and differentiated HFK and CIN612 cells were harvested. Resuspend the pellet in 5 volumes of CE buffer containing detergent [10 mM HEPES, 60 mM KCl, 1 mM EDTA, 0.075% (v/v) NP-40, 1 mM DTT, and 1 mM PMSF, adjusted to pH 7.6], incubate on ice for 3 min for cell lysis, then centrifuge at 1,000 to 1,500 rpm for 4 min. Collect the supernatant as the cytoplasmic fraction, wash the nuclear pellet three times with detergent-free CE buffer, then extract nuclear proteins by resuspending in nuclear extraction (NE) buffer [20 mM Tris Cl, 420 mM NaCl, 1.5 mM MgCl2, 0.2 mM EDTA, 1 mM PMSF, and 25% (v/v) glycerol, adjusted to pH 8.0] adjusted to 400 mM salt concentration and incubating on ice for 10 min with periodic vortexing. Immunoblotting was performed on the extracted Nuclear and cytoplasmic pools of proteins.

### siRNA Transfection.

Cells were seeded at a confluence of 30 to 40% in a 6-well plate, followed by transfection with siRNAs targeting E6AP (Dharmacon, SMARTPool no. L-005137-00-0005), custom si 18E6/E7 (Dharmacon, sequence 5’ CAUUUACCAGCCCGACGAG 3’), and siLuci as a control (Dharmacon, no. D-002050-0-1-20) at a final concentration of 50 nM using Lipofectamine RNAMaxi (Life Sciences Technologies, no. 13778-150). After 72 h of incubation, cells were harvested, lysed in 2× SDS lysis buffer, and subsequently analyzed by western blotting.

### Chromatic Immunoprecipitation Assay (CHIP).

Cells were cross-linked with 1% formaldehyde and collected in RIPA buffer. CHIP was performed on the cells using the protocol as previously described (44). The primers used for CHIP assay and RT-qPCR are listed below:

**Table:** 

Primer Name	Sequence 5’-3’
ALU FWD ALU REV	ACG AGG TCA GGA GAT CGA GA CTC AGC CTC CCA AGT AGC TG
URR FWD URR REV	GAT GCA GTA GTT CTG CGG TTT TAT GTT GGC AAG GTG TGT TAG G
E6 FWD E6 REV	GAC CTC GGA AAT TGC AAC ATG CTA TGC AAC GTC CTG
E7 FWD E7 REV	AAT TAC CCG ACA GCT CAG ATG GGC ACA CGA TTC CAA ATG AG
E1 FWD E1 REV	GAC AGA CAG ACA GGG G CCC GCT GTC TGG AAG TTC
E2 FWD E2 REV	TAC TGT TGT GGA AGG GCA AG TCC CAG CAA AGG ATA TTT CGT C
Late PolyA FWD Late PolyA REV	GCG TGT GTA CTT GTA GCA ACC GAA AAC GGT TAG G
Early PolyA FWD Early PolyA REV	GGT ATT GGT ATT GGT ATT GG ACC CAT ACT ACC ATA CCT TA
TOP1α FWD TOP1α REV	ATGAGCGGCGATCATCTGC TCTTTATGTTCGCTGTTGCTA
TOP3β FWD TOP3β REV	ATGAAAACCGTGCTGATGGTGG GCTGGTCATTTTAAAGCGCACC
TOP3α FWD TOP3α REV	TATCATCTGTATGGCCAGAACGTG TCCACAAAGTTTTCCGGGCAATA
MYADM FWD MYADM REV	CGT AGG TGC CCT AGT TGG GAG TCC ATT CTC ATT CCC AAA CC
EGR1 FWD EGR1 REV	GAA CGT TCA GCC TCG TTC TC GGA AGG TGG AAG GAA ACA CA

### Alkaline COMET Assay.

The cells underwent an alkaline COMET assay following the manufacturer’s protocol (Abcam, #ab238544). In summary, a base layer of COMET agarose was established on the slides. Cell samples were combined with agarose and applied as a secondary layer on top of the base. Cells were subsequently lysed with lysis buffer (60 min at 4 °C) and alkaline solution (30 min at 4 °C). Electrophoresis was conducted under alkaline conditions. DNA visualization was accomplished by staining with VISTA green DNA dye, and images were captured using an epifluorescence microscope. The tail movement of the damaged DNA was measured using the OpenCOMET plugin in ImageJ.

### Immunofluorescence.

For immunofluorescence staining, cells were seeded at a density of 1 × 10^^5 per^ well in a 4-chambered slide (MatTek). After 24 h of incubation, the cells were washed twice with PBS and then fixed with 4% formaldehyde for 20 min at room temperature. Following fixation, the cells were washed three times with PBS and permeabilized with 0.5% Triton X-100, followed by blocking with 1% BSA for 1 h at room temperature. The cells were incubated with primary antibodies (anti-rabbit TOP1α, 1:500; anti-rabbit TOP3α, 1:200; and anti-mouse TOP3β, 1:200). The cells were then washed three times with PBS and incubated with secondary antibodies (Alexa Fluor 564-conjugated anti-rabbit and mouse antibodies, 1:500) for 1 h in a humidified chamber at room temperature, followed by three 5-min washes with PBS. The cells were stained with DAPI and mounted. Image acquisition was performed using a Nikon Ti2B fluorescence microscope, and image analysis was done using ImageJ software.

For immunofluorescence analysis of HPV-positive paraffin-embedded tissue sections, paraffin-embedded slides were first deparaffinized using a series of xylene and ethanol washes. This process included 15-min washes with xylene, followed by a 5-min treatment with a 1:1 xylene and ethanol mixture at room temperature. Next, sequential ethanol washes were performed on the slides, consisting of two 2-min washes each with 100%, 95%, and 70% ethanol. After this, the cells were washed thrice with distilled water, followed by antigen retrieval by heating the slides at 60 °C overnight. Immunofluorescence staining on the slides was then conducted using the protocol described above.

### Southern Blot Analysis.

Total cellular DNA was extracted from CIN612 control and topoisomerase knockdown cells and digested with restriction enzymes to linearize episomal HPV DNA. The digested DNA samples were separated by agarose gel electrophoresis and transferred to nitrocellulose membranes. Membranes were hybridized with P32 radioactively labeled HPV31-specific probes to detect viral DNA forms in CIN612 cells. Following hybridization and washing steps, membranes were exposed to autoradiography film for the detection of radioactive signals. Band intensities were quantified using densitometry to determine relative levels of total viral DNA in knockdown versus control cells as outlined in previous studies (57).

### Western Blot Analysis.

Total cell extracts were obtained by lysing the cells directly in a 2× SDS-PAGE sample buffer. Western blotting and processing were then conducted as previously described. Image acquisition was carried out using the Odyssey Fc LiCor system.

### XTT-Based Cell Growth Assay.

Cell viability and growth pattern were assessed using the XTT colorimetric assay. SCR control CIN612 cells and topoisomerase knockdown cell lines (shTOP1α, shTOP3α, and shTOP3β) were seeded at 5,000 cells per well in 96-well plates in triplicate. Cell viability was measured at Days 1, 2, 3, 4, 6, and 8 postseeding by adding 50 μL of XTT reagent per well and incubating for 4 h at 37 °C (as per the manufacturer’s protocol, Thermo Scientific, #X12223). Absorbance was measured at 450 nm with 630 nm reference wavelength using a microplate reader. Growth curves were plotted using GraphPad Prism software, with all values normalized to SCR control to determine relative cell viability and proliferation rates.

### RT-qPCR Analysis.

Cells were collected, and RNA was extracted using Qiagen’s RNeasy mini-plus kit extraction protocol (Qiagen, # 74134) and subjected to reverse transcription using the high-capacity RNA to cDNA kit’s protocol (Thermo Fisher, #4387406). qPCR was performed using LightCycler 480 SYBR green I master mix (Thermo Fisher, # 4707516001). The primers used are listed in the CHIP protocol section.

### RADAR (Rapid Approach to DNA Adduct Recovery) Assay.

1 × 10^6^ cells were lysed using lysis buffer (6 M GTC, 10 mM Tris-HCl, pH 6.5, 20 mM EDTA, 4% Triton X-100, 1% Sarkosyl, and 1% DTT) for 30 min on ice, and DNA–protein cleaved complexes were isolated. Nucleic acids were recovered by adding ½ volume of 100% ethanol, followed by incubation for 30 min at −20 °C and centrifugation for 15 min at maximum speed. DNA was quantified, and an equal amount of DNA from each cell line was spotted onto the nitrocellulose membrane. The membrane was allowed to dry at room temperature, then blocked in 1% BSA before being subjected to immunoblot analysis.

### RNA-seq Analysis.

1.5 × 10^7^ cells were taken, and RNA was extracted using the manufacturer’s protocol (Qiagen, # 74134). Extracted RNA from each sample was sent to Northwestern University’s Core sequencing facility to generate sequenced fastq files. BGI Genomics analyzed the data. The RNA-seq data generated in this study have been deposited in the NCBI Gene Expression Omnibus (GEO) database under accession number GSE312527.

### S9.6 Dot Blot Analysis.

R-loops were detected by performing S9.6 dot blot analysis on the cells using the protocol described in previous studies (44). In brief, cell lysates were subjected to phenol-chloroform DNA extraction, were spotted onto the Zeta-probe membrane, and then blocked with 5% BSA in TBST. The membrane was probed overnight at 4 °C with the S9.6 anti-RNA: DNA hybrid antibody, followed by secondary antibody incubation and ECL development (Fisher, 4500085). Image acquisition was then performed using an Odyssey Fc LiCor.

### DRIP-qPCR (DNA: RNA Immunoprecipitation) Analysis.

DRIP-qPCR was conducted on cells following the previously described protocol (63). Briefly, DNA was extracted from cells using the phenol-chloroform extraction method after an overnight treatment with RNase A (5 ng/mL) and Proteinase K (7.5 ng/mL) at 37 °C. After shearing the DNA through Bioruptor or mung bean nuclease digestion, samples underwent immunoprecipitation with RNA: DNA hybrid antibody on magnetic beads. Following several wash steps, the DNA was eluted, purified using a PCR purification kit, and stored at −20 °C. The primers used for analysis are listed in the CHIP protocol section.

### Statistical Analysis.

All experiments were conducted at least three times, and the data are presented as the mean and SD of the mean. Statistical significance was assessed using GraphPad Prism software with the student’s unpaired two-tailed *t* test and two-way ANOVA. A *P* value below 0.05 was deemed statistically significant, and throughout the text, the *P* values are defined as follows: *P < 0.05; **P < 0.005; ***P < 0.001; “ns” indicates a nonsignificant *P* value greater than 0.05.

The band intensities of Western blots were measured using ImageJ software to quantify protein levels. The final relative quantification values are the ratio of the net band to the net loading control bands of GAPDH or nucleolin. All experiments and statistics are performed from three independent replicates.

## Supplementary Material

Appendix 01 (PDF)

Dataset S01 (CSV)

Dataset S02 (CSV)

## Data Availability

The RNA sequencing data, including FASTQ files and metadata, have been deposited in the NCBI Gene Expression Omnibus (GEO) under accession GSE312527 (64). Study data are included in the article and/or supporting information.
