# Supplementary material for: Differential roles of type I topoisomerases in regulating HPV pathogenesis
Source: Proc Natl Acad Sci U S A. 2026 Jan 2;123(1):e2526296123. doi: 10.1073/pnas.2526296123 (PMC12773723; doi:10.1073/pnas.2526296123)
Supplement: Supplementary file 1 — Appendix 01 (PDF) [file pnas.2526296123.sapp.pdf]

Differential Roles of Type I Topoisomerases in Regulating HPV Pathogenesis

Arushi Vats, Conor W. Templeton, and Laimonis Laimins*

Dept of Microbiology-Immunology, Northwestern University, Chicago, IL 60611

*Corresponding author: Laimonis Laimins
l-laimins@northwestern.edu

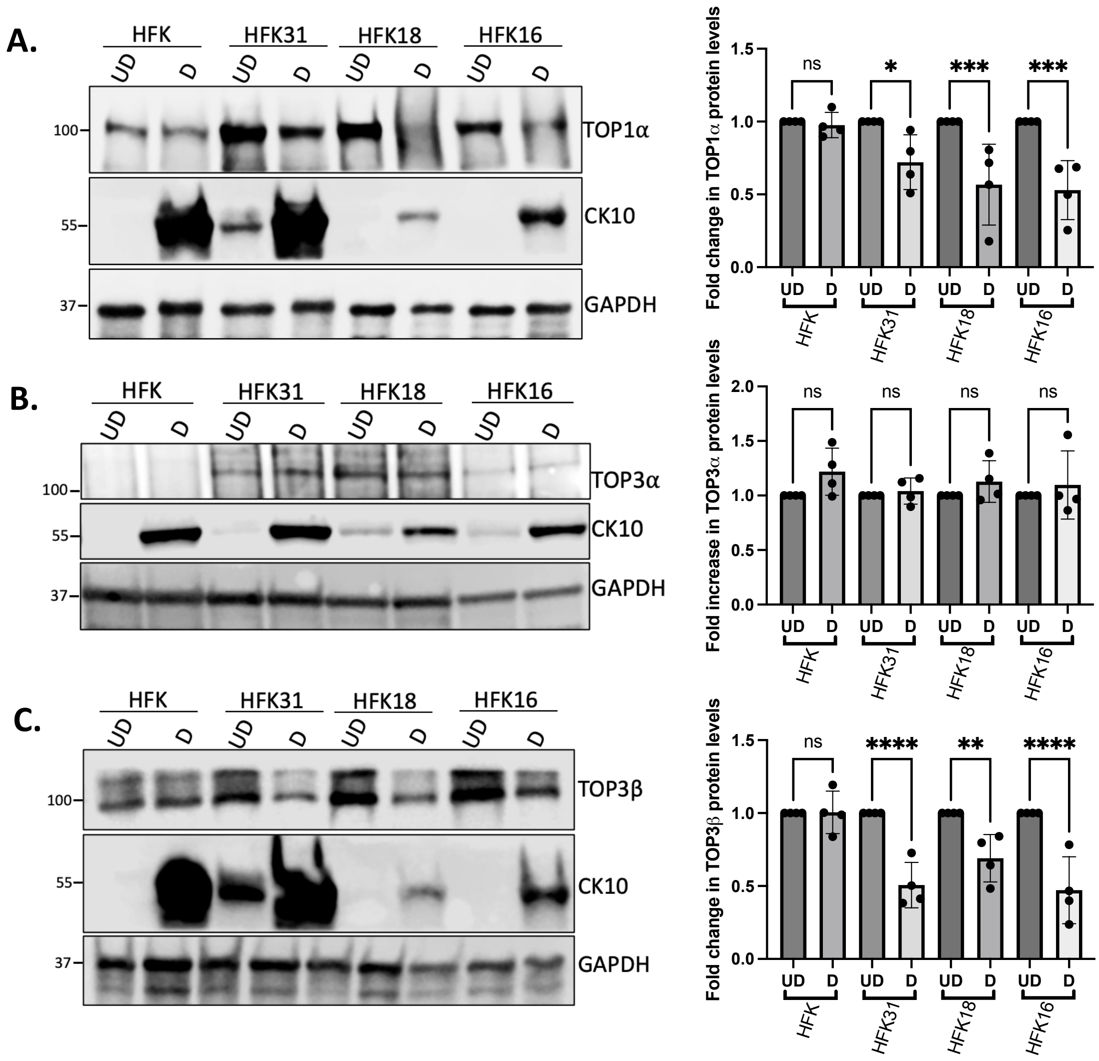

Fig. S1. Regulation of topoisomerases during keratinocyte differentiation in HFKs control, HPV-31, 18, and 16 positive HFKs: (A-C) Western blot analysis displaying protein levels of TOP1α (A), TOP3α (B), and TOP3β (C) in undifferentiated (UD) and differentiated (D) keratinocytes that maintain HPV31, 18, or 16 episomes and HFKs control. CK10 acts as a differentiation marker, and GAPDH serves as a loading control. The quantification of protein levels normalized to undifferentiated samples is presented on the right. Statistical significance: ns (not significant), *p < 0.05, **p < 0.01, ***p < 0.001. All experiments and statistics are performed from three independent replicates.

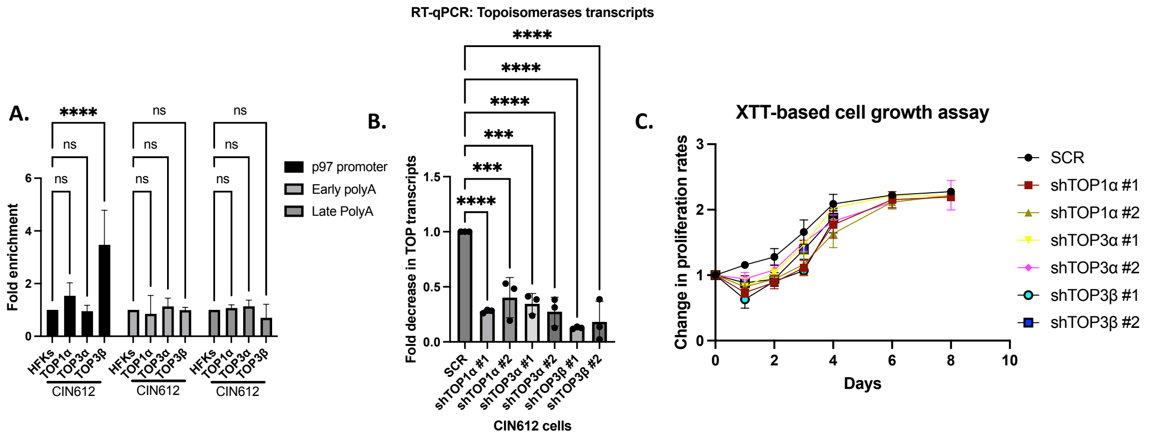

Fig. S2. Characterization of topoisomerase binding, knockdown efficiency and growth effects in HPV-positive cells. (A) ChIP analysis showing enrichment of topoisomerases at viral genomic regions: Fold enrichment of TOP1α, TOP3α, and TOP3β binding at the p97 promoter, early polyA, and late polyA regions in HFK and CIN612 cells relative to IgG control. (B) RT-qPCR analysis demonstrating knockdown efficiency: Transcript levels of TOP1α, TOP3α, and TOP3β in CIN612 cells transduced with two independent shRNA constructs (#1 and #2) targeting each topoisomerase, normalized to scrambled control (SCR). (C) XTT-based cell viability assay: Growth curves showing proliferation rates of scrambled control (SCR) and topoisomerase knockdown CIN612 cell lines over 8 days. Data points represent fold change in proliferation rates relative to Day 0. Each curve represents cells transduced with independent shRNA constructs (#1 and #2) for each topoisomerase. Data represent the mean ± SD from three independent experiments.

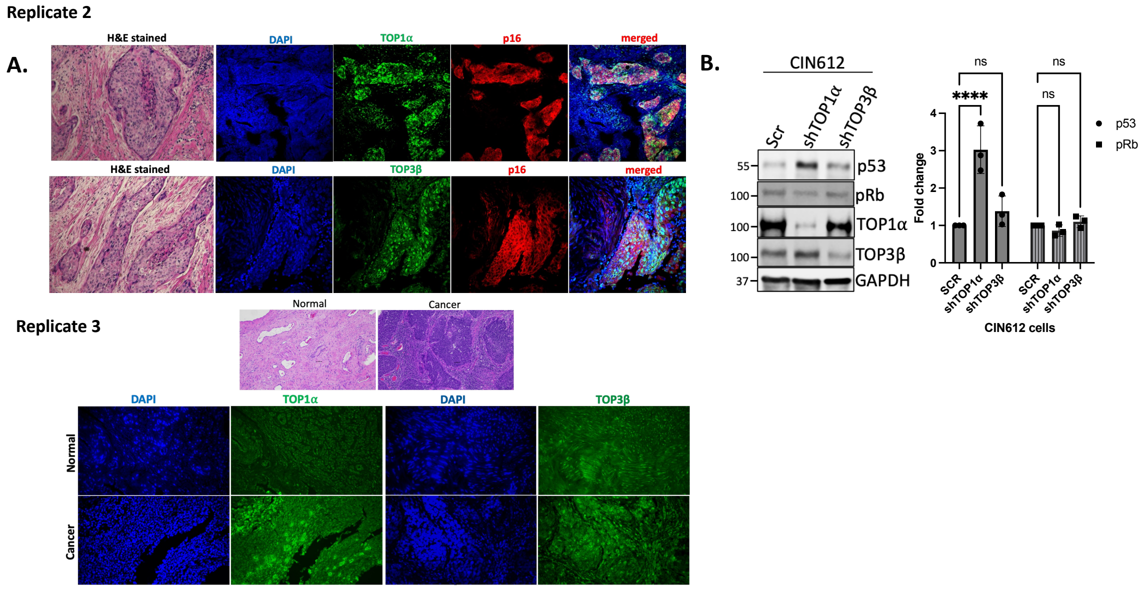

Fig. S3. Immunofluorescence analysis of TOP1α and TOP3β expression in cervical tissue specimens (A). Representative images showing H&E staining (left) and immunofluorescence analysis of normal cervical tissue (top panels) and HPV-positive cervical cancer tissue (bottom panels) from two independent patient samples (Replicate 2 and Replicate 3). Tissues were stained for TOP1α or TOP3β (green), p16INK4a (red), and DAPI (blue nuclei). Merged images show co-localization patterns. Scale bars indicate magnificatio**n.** (B) Analysis of p53 and pRb protein levels in topoisomerase-depleted CIN612 cells. Western blot analysis showing p53, pRb, TOP1α, and TOP3β protein levels in scrambled control (SCR) and topoisomerase knockdown CIN612 cells (shTOP1α and shTOP3β). GAPDH serves as loading control. Quantification graph (right) shows fold change in protein levels normalized to GAPDH and SCR control. Statistical significance: ns (not significant), ****$p < 0.00001$. All experiments and statistics are performed from three independent replicates.

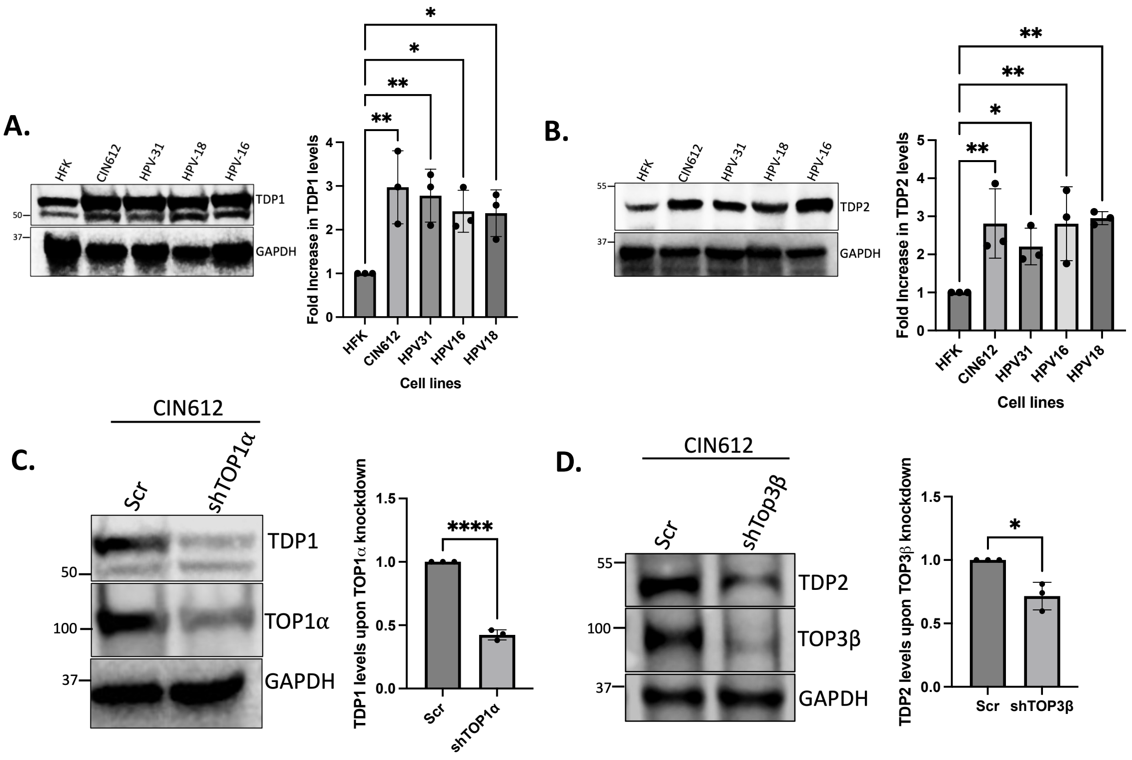

Fig. S4. Analysis of TDP1 and TDP2 expression in HPV-positive cells: (A-B) Western blot analysis and quantification of TDP1 (A) and TDP2 (B) protein levels in HFK, CIN612, and HPV31/16/18-positive cells. (C) Western blot showing TDP1 levels in control (Scr) and TOP1α knockdown CIN612 cells with quantification. (D) Western blot showing TDP2 levels in control (Scr) and TOP3β knockdown CIN612 cells with quantification. GAPDH serves as loading control. Statistical significance: *p < 0.05, **p < 0.01, ****p < 0.0001. Data shown are from three independent experimental replicates with statistical analysis performed accordingly.

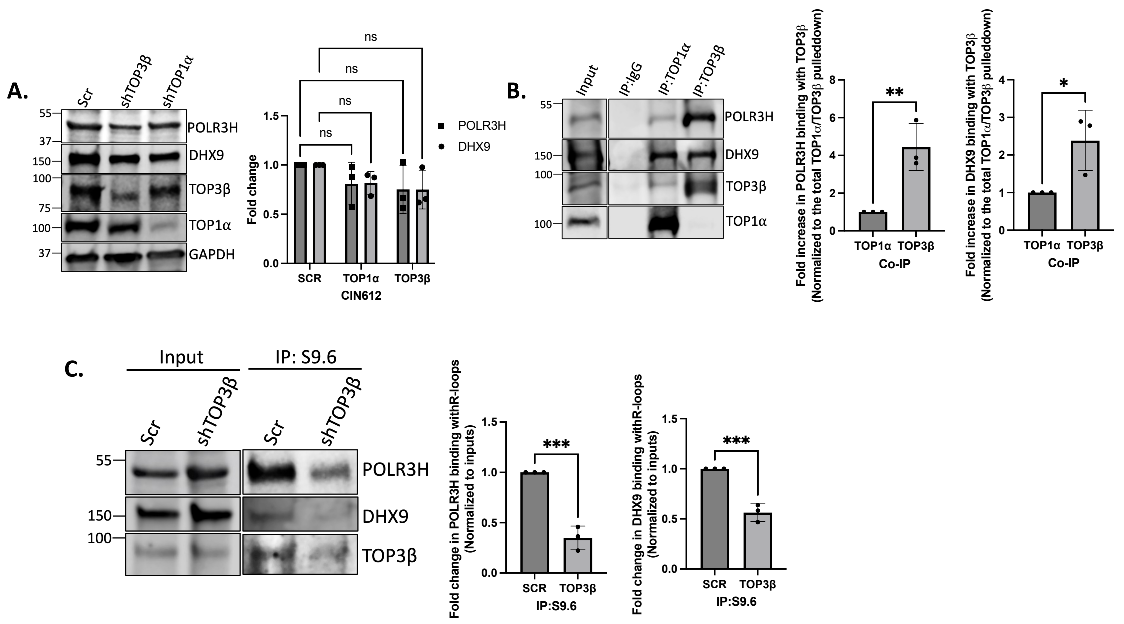

Fig. S5. Analysis of TOP3β-interacting proteins. (A) A Western blot demonstrating the protein levels of POLR3H, DHX9, TOP3β, and TOP1α in control and knockdown cells. (B) Co-immunoprecipitation analysis illustrating the interaction of TOP1α and TOP3β with POLR3H and DHX9. (C) S9.6 immunoprecipitation displaying the recruitment of POLR3H and DHX9 to R-loops in control and TOP3β or TOP1α knockdown cells. The input serves as a loading control. Results are representative of three independent biological replicates.
